# Staging laparoscopy for gastric cancer: European consensus

**DOI:** 10.1093/bjs/znaf144

**Published:** 2025-09-05

**Authors:** Sander J M van Hootegem, Niels A D Guchelaar, Karen van der Sluis, Lianne Triemstra, Stefan P Mönig, Karol Rawicz-Pruszyński, Riccardo Rosati, Paolo Morgagni, Maria Erodotou, Leonardo Solaini, Giovanni De Manzoni, Wojciech Polkowski, Francesco Puccetti, Simone Giacopuzzi, Suzanne S Gisbertz, Jimmy B Y So, Jelle P Ruurda, Pieter S L van der Sluis, Sjoerd M Lagarde, Johanna W van Sandick, Bas P L Wijnhoven, Other Members of the IGCA European Chapter Collaborative Group on Staging Laparoscopy for Gastric Cancer, Alexander A F A Veenhof, Adam Zeyara, Alan Patrick Ainsworth, Alessia Malagnino, Alexander W Phillips, Anastasios Kottikias, Andrew R Davies, Arto Kokkola, Bo J Noordman, Carlo Vallicelli, Carolina Canhoto, Cecilia Monteiro, Claudia Neves Marques, Claudio Belluco, Daniel Reim, Daniele Marrelli, Daromir Godula, David James, Davide Zattoni, Dimitrios Schizas, Dimitrios Theodorou, Dionysios Dellaportas, Eelco B Wassenaar, Eider Talavera-Urquijo, Fabio Uggeri, Fabrizio D’Acapito, Fausto Rosa, Federica Riccio, Francesco Abboretti, Geert A Simkens, Gianluca Garulli, Gianmario Edoardo, Giovanni de Manzoni, Grard A P Nieuwenhuijzen, Guido A M Tiberio, Henk H Hartgrink, Hanne Vanommeslaeghe, Hylke J F Brenkman, Ines Gockel, Ioannis G Karavokyros, Ioannis Rouvelas, J W Haveman, Jacopo Weindelmayer, Jan Willem T Dekker, Jessie A Elliott, Joos Heisterkamp, Jose A L Barbosa, José P Freire, Koen J Hartemink, Lapo Bencini, Lieven P Depypere, Luca Provenzano, Lucia Moletta, Ludovico Carbone, Luigina Graziosi, Mark I van Berge Henegouwen, Magnus Nilsson, Marcel A Schneider, Marco Milone, Maria Bencivenga, Marloes Emous, Mattia Berselli, Mauro Zago, Meindert N Sosef, Michael Hareskov Larsen, Michele Manara, Michele Valmasoni, Michiel F G de Maat, Monica Gualtierotti, Monica Miro Martin, Fahad Mahmood, Nezih Akkapulu, Paolo Morgagni, Paolo Parise, Paul A Carroll, Paul M Schneider, Pedro Azevedo Serralheiro, Ines Gockel, Radoslaw Pach, Raffaele De Luca, Renato Salvador, Renol M Koshy, Rita Alfieri, Sara Pollesel, Markus Schäfer, Sheraz R Markar, Silvia Ministrini, Stefan Antonowicz, Stefania A Piccioni, Stefano Olmi, Stefano Rausei, Styliani Mantziari, Tania Triantafyllou, Victor Turrado-Rodriguez, Wendy Kelder, Yannick Deswysen, Yves Borbély

**Affiliations:** Department of Surgery, Erasmus Medical Centre, Rotterdam, The Netherlands; Department of Medical Oncology, Erasmus MC Cancer Institute, Rotterdam, The Netherlands; Department of Surgery, Antoni van Leeuwenhoek, Amsterdam, The Netherlands; Department of Surgery, University Medical Centre Utrecht, Utrecht, The Netherlands; Division of Digestive Surgery, University Hospital of Geneva, Geneva, Switzerland; Department of Surgical Oncology, Medical University of Lublin, Lublin, Poland; Department of Gastrointestinal Surgery, IRCCS San Raffaele Scientific Institute, Milan, Italy; Department of General Surgery, Morgagni-Pierantoni Hospital, Forlì, Italy; Department of Medical and Surgical Sciences, University of Bologna, Forlì, Italy; Department of Surgery, Erasmus Medical Centre, Rotterdam, The Netherlands; Department of Medical and Surgical Sciences, University of Bologna, Forlì, Italy; Upper G.I. Surgery Division, University of Verona, Verona, Italy; Department of Surgical Oncology, Medical University of Lublin, Lublin, Poland; Department of Gastrointestinal Surgery, IRCCS San Raffaele Scientific Institute, Milan, Italy; Department of Medical and Surgical Sciences, University of Bologna, Forlì, Italy; Department of Surgery, Amsterdam UMC location University of Amsterdam, Amsterdam, The Netherlands; Cancer Treatment and Quality of Life, Cancer Centre Amsterdam, Amsterdam, The Netherlands; Department of Surgery, Yong Loo Lin School of Medicine, National University of Singapore, Singapore; Department of Surgery, University Medical Centre Utrecht, Utrecht, The Netherlands; Department of Surgery, Erasmus Medical Centre, Rotterdam, The Netherlands; Department of Surgery, Erasmus Medical Centre, Rotterdam, The Netherlands; Department of Surgery, Antoni van Leeuwenhoek, Amsterdam, The Netherlands; Department of Surgery, Erasmus Medical Centre, Rotterdam, The Netherlands

## Introduction

Gastric cancer is the fifth most prevalent cancer and the third most common cause of cancer-related deaths globally^1^. Gastric cancer frequently metastasizes to the peritoneal cavity. The incidence of peritoneal metastases at the time of diagnosis, including free cancer cells in peritoneal washings, ranges from 15% to 32%, and its presence is associated with a poor prognosis^2,3^. Due to the low sensitivity of conventional imaging such as CT or PET-CT for peritoneal metastases, staging laparoscopy is performed to exclude small-volume macroscopic peritoneal dissemination or free cancer cells, and to assess the local resectability of the tumour^4–6^.

Although staging laparoscopy has been part of diagnostic workup for patients with gastric cancer for over a decade, guidelines differ on patient selection and criteria of those at risk of peritoneal dissemination. Several guidelines limit its use to cT3/T4 tumours or gastric cancer with a high nodal burden (see *supplementary material S3*). In contrast, the National Institute for Health and Care Excellence (NICE) guideline recommends performing staging laparoscopy in all patients with gastric cancer^7^. Interestingly, all guidelines lack recommendations on how to perform staging laparoscopy, likely due to the significant variation in technical performance, which complicates comparison of procedure-based outcomes^8^. This inconsistency poses challenges when evaluating rates of peritoneal dissemination, limits the ability to understand the diagnostic accuracy of staging laparoscopy, and may lead to stage migration^9^. Standardizing staging laparoscopy would result in a more systematic execution of this diagnostic test, potentially leading to standardized staging and better patient selection, while improving data collection on clinical staging. It would facilitate international studies on gastric cancer staging and treatment of peritoneal metastases.

A Delphi study could drive standardization, as it provides a method to systematically achieve expert consensus, without overpowering opinions of dominant participants^10^. This approach is particularly appropriate for the present situation given the high heterogeneity in used techniques, largely influenced by individual surgeons’ preferences^8^. Therefore, the authors performed a modified Delphi study to examine the extent of practice variation of staging laparoscopy amongst European gastric cancer surgeons, aiming to establish a standardized protocol.

## Methods

### Study design and participant recruitment

A modified Delphi study, involving a steering committee, was conducted between August 2023 and April 2024^11^. The steering committee, composed of experienced upper-gastrointestinal (GI) or gastric cancer surgeons from various European countries (the authors) invited by the senior author (B.P.L.W.), coordinated the study. The project leaders from a recent Dutch Delphi consensus were part of the steering committee^12^. To recruit expert participants, the Dutch Upper GI Cancer Group (DUCG), the European Society for Diseases of the Esophagus (ESDE), the European chapter of the International Gastric Cancer Association (IGCA), and the Italian Research Group for Gastric Cancer (GIRCG) were approached for endorsement and to promote this Delphi study through their networks. This strategy was chosen to ensure inclusion of participants with a high level of expertise from diverse geographical and institutional backgrounds and to optimize engagement across Delphi rounds. An invitation for participation was sent out through these networks, targeting European surgeons practicing upper-GI or gastric cancer surgery who perform staging laparoscopies for gastric cancer. Participants were asked to provide contact information and fill out the first survey. Sole specialization in upper-GI or gastric cancer surgery was not mandatory to avoid excluding surgeons from general hospitals. As this study did not involve patient or privacy-sensitive data, review and registration by the medical ethics committee were not necessary. The results are reported according to the ACCORD checklist^13^.

### Development of the survey

A systematic review was performed to assess pertinent aspects of staging laparoscopy, which was published recently^8^. Additionally, a review of the current guidelines was performed to assess variation in indications for staging laparoscopy (see *supplementary material S3*). These parameters provided input for the survey developed by the study team (K.v.d.S., L.T., N.A.D.G. and S.J.M.v.H.), covering five domains: indications for staging laparoscopy, assessment of resectability, inspection of the peritoneal cavity, peritoneal lavage and biopsy, and re-laparoscopy. The survey was reviewed and approved by the steering committee, who could suggest adaptations or propose new statements based on their expertise. Surgical access, choice of equipment, and patient position were topics considered outside the scope of this study.

### Modified Delphi method

The study consisted of two predetermined survey rounds conducted using Castor EDC software version 2023.3.0.1 (Castor, Amsterdam, The Netherlands), sent out digitally^14^. All responses were processed anonymously. Evidence was not presented to participants before or during the survey. In the first round, a three-point Likert scale (agree/neutral/disagree) was used per statement, followed by a two-point Likert scale (agree/disagree) in subsequent rounds, along with feedback on previous results. The subsequent surveys were sent out to all who completed the first round. Free text comments were solicited. The results of each round were discussed during an online meeting with the steering committee, clustered per domain. Statements without consensus or with <80% agreement were discussed and rephrased or adapted when unclear based on comments by the participants or steering committee. After adaptation, the steering committee screened and approved the survey before sending it to participants. In case consensus was reached on a statement, it was included in the following round to prove the stability of results. If a third round was deemed necessary for achieving consensus or would prove stability of results, statements meeting these criteria in the first two rounds were excluded from this final round. Consensus was defined a priori as ≥70% agreement among participants, which is a commonly used threshold in Delphi studies^15^. This definition was communicated with participants at the start of the Delphi process. Participants were given 3 weeks to complete each survey. Up to three reminders were sent to non-respondents. Participants were provided the option to be identified as collaborators.

## Results

### Course of Delphi rounds

**Fig. 1:**
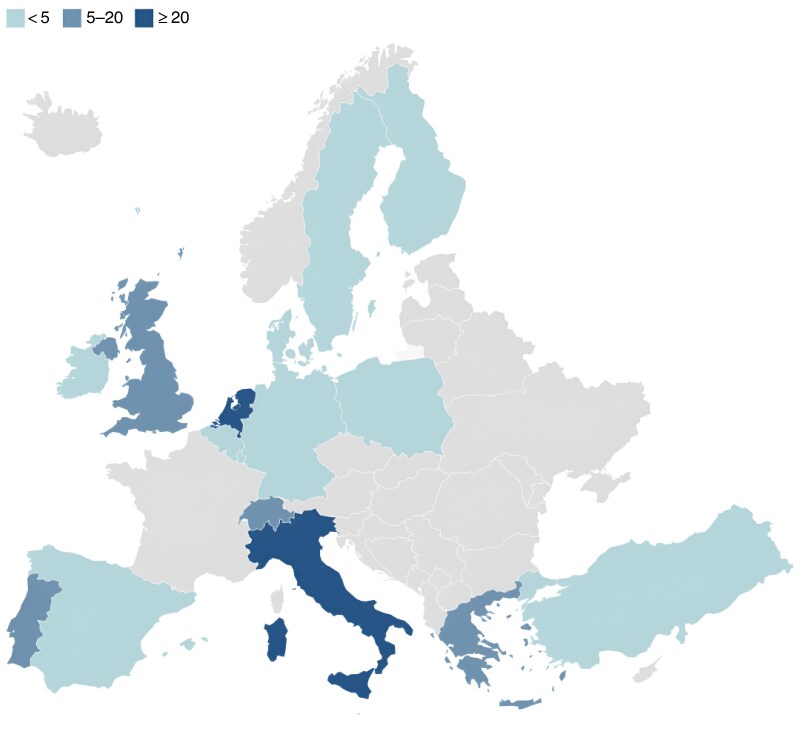
Survey participants per country

The first survey (November 2023) was completed by 111 respondents from 16 European countries (*Fig. 1*). The use of networks to recruit participants prevented calculation of the response rate or characterization of non-respondents. Among respondents, 79.3% (88 of 111) worked in a university hospital and 40.5% (45 of 111) performed >50 staging laparoscopies for gastric cancer annually. About half of the respondents (54.1%; 60 of 111) had a protocol for staging laparoscopy available at their hospital.

**Fig. 2:**
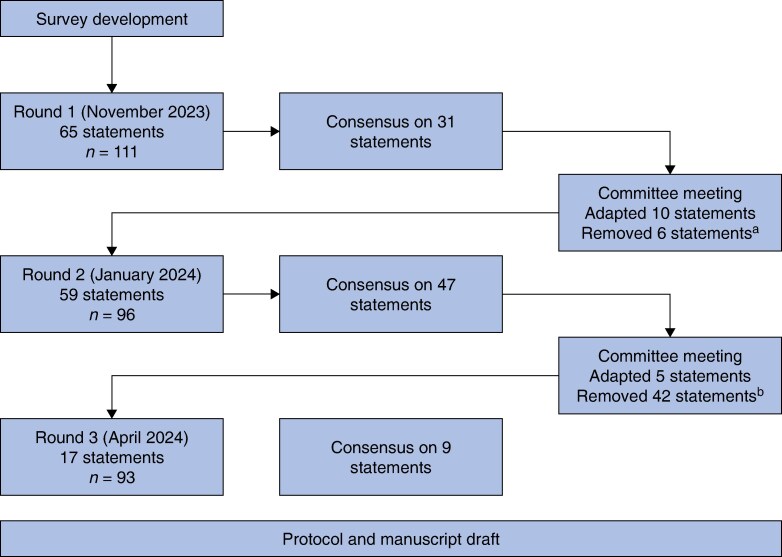
Flow chart of the Delphi process ^a^Statements on resectability (see *supplementary material S2*). ^b^All statements with stable results.

The response rate for the second round (January 2024) was 86.5% (96 of 111). The steering committee opted for a third round (April 2024), aiming to achieve consensus on resectability and re-laparoscopy, and prove stability on results concerning the indications for Siewert type II tumours. This final round had a response rate of 83.7% (93 of 111). See *Fig. 2* for a flow chart of the Delphi process. Statements that were removed are listed in *supplementary material S2*.

### Indications for staging laparoscopy

Statements on indications were divided into tumour characteristics and tumour location (according to Siewert classification for junctional cancers and anatomical region for gastric cancer) (*Table 1*)^16^. There was no consensus on whether all patients planned for curative treatment should undergo staging laparoscopy, irrespective of neoadjuvant chemotherapy or planned primary surgical resection. However, staging laparoscopy should be performed in patients with an increased risk of peritoneal metastases. This included patients with cT3–4 tumours, lymph node metastases (cN+), poorly cohesive tumours, and radiological suspicion of peritoneal metastases.

**Table 1:** Statements related to indications for staging laparoscopy

Statement	Consensus achieved?	Agree/disagree	Percentage	Stable results?
**Tumour characteristics**				
All patients undergoing curative primary surgical resection (not endoscopic) at the start of the planned resection.	No	Disagree	50.5	No
All cT3–4 gastric carcinoma who are scheduled for curative-intent treatment.	Yes	Agree	93.8	Yes
All cN+ gastric carcinoma who are scheduled for curative-intent treatment.	Yes	Agree	83.3	Yes
All patients with poorly cohesive gastric carcinoma who are scheduled for curative-intent treatment (including surgery).	Yes	Agree	76.0	Yes
All patients with gastric carcinoma and a high-risk profile of peritoneal metastases (poorly cohesive carcinoma and/or cT3–4 and/or cN+).	Yes	Agree	93.8	Yes
All patients with gastric carcinoma and radiological suspicion of peritoneal metastases.	Yes	Agree	90.6	Yes
**Tumour location**				
The indication for a staging laparoscopy is determined by the location of the primary tumour in the stomach (GOJ/proximal/distal).	No	Agree	52.0	No
Siewert type 3 tumour is an indication for a staging laparoscopy.	Yes	Agree	89.5	Yes
Siewert type 2 tumour is an indication for a staging laparoscopy.	No	Agree	59.4	Yes
Siewert type 1 tumour is an indication for a staging laparoscopy.	Yes	Disagree	88.5	Yes
Tumours with the bulk located in the stomach, irrespective of the length of oesophageal infiltration, are an indication for a staging laparoscopy.	Yes	Agree	86.5	Yes
In Siewert type 2 tumours, as seen on endoscopy, in which the PET-CT shows more involvement of the cardia/proximal stomach, a staging laparoscopy is indicated.	Yes	Agree	86.0	Yes
In Siewert type 2 tumours, as seen on endoscopy, in which the PET-CT shows more involvement of the oesophagus, a staging laparoscopy is indicated.	Yes	Disagree	73.1	No
For Siewert type 2 tumours at the junction, the indication for a staging laparoscopy is also determined by tumour characteristics such as advanced tumour or nodal stage.	Yes	Agree	83.9	No

Underlined text was rephrased during the course of the rounds. Statements are displayed as used in the final survey round they were included. GOJ, gastro-oesophageal junction.

There was consensus that when endoscopy shows a Siewert type I tumour, staging laparoscopy is not required, while for Siewert type III tumours staging laparoscopy is indicated. For Siewert type II tumours, as seen on endoscopy, participants agreed that staging laparoscopy is indicated when (PET-)CT shows more involvement of the proximal stomach. Importantly, the aforementioned high-risk features also determine the indication and must be considered in these tumours.

### Assessment of resectability

No consensus was achieved on whether resectability of the primary tumour should routinely be assessed during staging laparoscopy (*Table 2*). The first survey included statements on specific structures or organs that should be assessed (oesophagus and/or diaphragm, liver, spleen, mesocolon, pancreas, vascular structures posterior of the stomach, and the retroperitoneum). However, no consensus was reached regarding routine assessment or assessment when suspicious for involvement. Based on the participant comments and discussions within the steering committee, subsequent rounds divided structures into those requiring surgical dissection and those that do not. Consensus was reached that structures requiring dissection for assessment should only be evaluated if involvement is suspected based on imaging. Additionally, in such cases, the omental bursa should be opened to assess infiltration for tumours located on the posterior wall of the stomach.

**Table 2:** Statements related to assessment of resectability

Statement	Consensus achieved?	Agree/disagree	Percentage	Stable results?
The resectability of the primary tumour and/or the lymph nodes should be assessed during the staging laparoscopy.	No	Agree	67.7	Yes
All surrounding structures for which no further dissection is necessary (liver, spleen, etc.) should be assessed by standard for determining resectability.	No	Agree	64.6	Yes
All surrounding structures for which no further dissection is necessary (liver, spleen, etc.) should be assessed only when preoperative imaging is suspicious for involvement to determine resectability.	No	Disagree	53.1	Yes
All surrounding structures for which further dissection (pancreas, etc.) is necessary should be assessed by standard for determining resectability.	Yes	Disagree	77.4	Yes
All surrounding structures for which further dissection (pancreas, etc.) is necessary should only be assessed to determine resectability when preoperative imaging is suspicious for involvement.	Yes	Agree	75.0	Yes
The omental bursa should be opened to assess resectability in case the tumour is located at the posterior side of the stomach and infiltration is suspected on preoperative imaging.	Yes	Agree	82.3	Yes

Statements are displayed as used in the final survey round they were included.

### Inspection of the peritoneal cavity

Systematic inspection and documentation using the Peritoneal Cancer Index (PCI) of Sugarbaker should be performed during every staging laparoscopy^17^. Relevant structures that should be assessed include the greater and lesser omentum, small intestine (mesentery), hepatoduodenal ligament, pelvis, pouch of Douglas, and ovaries (*Table 3*).

**Table 3:** Statements related to inspection of the peritoneal cavity

Statement	Consensus achieved?	Agree/disagree	Percentage	Stable results?
The abdomen should be systematically inspected as a standard procedure.	Yes	Agree	95.8	Yes
The abdomen should be systematically inspected only when there is preoperative suspicion of peritoneal metastases.	Yes	Disagree	90.6	Yes
Inspection is performed according to the regions of the Peritoneal Cancer Index (PCI) by Sugarbaker *et al*.	Yes	Agree	92.7	Yes
Inspection is performed according to the Japanese Classification for Peritoneal Metastases (P1–P3).	Yes	Disagree	79.2	Yes
The greater omentum should be inspected as a standard procedure.	Yes	Agree	96.9	Yes
The greater omentum should only be inspected in cases of preoperative suspicion of peritoneal metastases.	Yes	Disagree	92.7	Yes
The lesser omentum should be inspected as a standard procedure.	Yes	Agree	92.7	Yes
The lesser omentum should only be inspected in cases of preoperative suspicion of peritoneal metastases.	Yes	Disagree	92.7	Yes
(The mesentery of) the small intestine should be inspected as a standard procedure.	Yes	Agree	80.2	Yes
(The mesentery of) the small intestine should only be inspected in cases of preoperative suspicion of peritoneal metastases.	Yes	Disagree	77.1	Yes
The hepatoduodenal ligament should be inspected as a standard procedure.	Yes	Agree	84.4	Yes
The hepatoduodenal ligament should only be inspected in cases of preoperative suspicion of peritoneal metastases.	Yes	Disagree	82.3	Yes
The pelvis should be inspected as a standard procedure.	Yes	Agree	94.8	Yes
The pelvis should only be inspected when there is preoperative suspicion of peritoneal metastases.	Yes	Disagree	91.7	Yes
The cavum Douglasi should be inspected as a standard procedure.	Yes	Agree	91.7	Yes
The cavum Douglasi should only be inspected when there is preoperative suspicion of peritoneal metastases.	Yes	Disagree	88.5	Yes
The ovaries should be inspected as a standard procedure.	Yes	Agree	84.4	Yes
The ovaries should only be inspected when there is preoperative suspicion of peritoneal metastases.	Yes	Disagree	82.3	Yes

Statements are displayed as used in the final survey round they were included.

### Peritoneal lavage and biopsy

Peritoneal lavage for cytological assessment should be performed in all patients (*Table 4*) undergoing staging laparoscopy. Free fluid (ascites) should be collected if present, but, when an insufficient amount (that is <50 ml) is aspirated, peritoneal lavage should also be performed. Preferred sites for peritoneal lavage were the right and left upper abdomen and the pouch of Douglas (see *supplementary material S4*), in agreement with the AJCC staging manual^18^. In the second survey, consensus was reached to perform lavage as follows: ≥200 ml should be instilled in the upper right and left subphrenic spaces and the pouch of Douglas; and ≥50 ml should be aspirated for cytological assessment. No consensus was reached on whether peritoneal lavage should be performed in patients who undergo primary surgical resection (that is not receiving neoadjuvant chemotherapy). During the study, an inventory of cytological processing methods was conducted by distributing questions to the pathologists of the steering committee centres (see *supplementary material S5*).

**Table 4:** Statements related to peritoneal lavage and biopsy

Statement	Consensus achieved?	Agree/disagree	Percentage	Stable results?
**Peritoneal lavage**				
Free fluid in the abdominal cavity (ascites) should be collected for cytology as a standard procedure.	Yes	Agree	99.0	Yes
Peritoneal lavage fluid should be collected for cytology as a standard procedure.	Yes	Agree	93.8	Yes
Peritoneal lavage fluid should be left in the abdomen without pneumoperitoneum for a few minutes before re-insufflating and aspirating the sample.	No	Disagree	69.8	Yes
According to the AJCC guideline, peritoneal washing should consists of instilling ∼200 cc of normal saline into the right and left subphrenic space and the pouch of Douglas. Ideally, >50 cc of washings should be retrieved for cytological assessment.	Yes	Agree	90.3	Yes
When a sufficient amount (i.e. >50 cc) of existing ascites has already been aspirated for cytological evaluation, peritoneal lavage fluid should be collected.	No	Disagree	63.4	Yes
When no sufficient amount (i.e. <50 cc) of existing ascites has already been aspirated for cytological evaluation, peritoneal lavage fluid should be collected.	Yes	Agree	91.7	Yes
In patients who will undergo a primary surgical resection, peritoneal lavage for cytological assessment should be performed in all patients given its prognostic value.	No	Agree	53.8	Yes
In patients planned for primary surgical resection, peritoneal lavage should be performed separately prior to planned primary surgical resection because positive cytology may change plan of management.	No	Disagree	62.4	Yes
In patients who undergo a primary surgical resection, peritoneal lavage should be performed when macroscopic metastases are absent during primary surgical resection.	No	Disagree	57.0	Yes
**Taking biopsies**				
All suspected abnormalities for peritoneal metastases should be biopsied.	No	Agree	60.4	Yes
A biopsy should be performed from all regions with suspect abnormalities for peritoneal metastases.	No	Agree	57.3	Yes
Suspicion of peritoneal metastases should be proven in at least one region.	Yes	Agree	93.8	Yes
A biopsy should be performed from all regions with abnormalities in case of suspected limited peritoneal disease.	Yes	Agree	74.0	Yes
A biopsy should be performed from all regions with abnormalities in case of suspected extensive peritoneal disease.	Yes	Disagree	86.5	Yes
A biopsy should be performed from one region with abnormalities in case of suspected extensive peritoneal disease.	Yes	Agree	86.5	Yes
Taking multiple biopsies should only be performed in case of suspected extensive peritoneal disease when it has consequences for patient management/treatment.	Yes	Agree	90.6	Yes
Suspicion of peritoneal metastases should be noted in the OR report.	Yes	Agree	99.0	Yes
The PCI score/*P*-value should be noted as a standard procedure in the OR report.	Yes	Agree	95.8	Yes

Underlined text was rephrased during the course of the rounds. Statements are displayed as used in the final survey round they were included. cc, ml; OR, operation report; PCI, Peritoneal Cancer Index.

Not all suspected abnormalities of the peritoneum require biopsy, but disease should be confirmed in at least one region. In case of limited disease, all regions should be biopsied; in case of extensive disease, biopsies of one region are sufficient unless taking multiple biopsies would impact choice of treatment.

### Re-laparoscopy

No consensus was reached whether routine restaging laparoscopy prior to surgery should be performed, nor on wether with it should involve a systematic or a global inspection (*Table 5*). However, staging laparoscopy is indicated in case of tumour progression on radiological imaging, but should not necessarily be planned separately from the intended surgical resection.

**Table 5:** Statements related to re-laparoscopy

Statement	Consensus achieved?	Agree/disagree	Percentage	Stable results?
After a negative laparoscopy *, the abdomen should be inspected again systematically (with PCI score) for peritoneal metastases as a standard procedure at time of the planned resection.	No	Disagree	53.8	Yes
After a negative laparoscopy *, the abdomen should be inspected again globally (without PCI score) for peritoneal metastases as a standard procedure at time of the planned resection.	No	Agree	54.8	Yes
Prior to the planned gastric resection, the abdomen should be inspected again systematically for peritoneal metastases in case of tumour progression.	Yes	Agree	89.6	Yes
A restaging laparoscopy should always be planned separately from the planned gastric resection.	Yes	Disagree	81.7	Yes

Underlined text was rephrased during the course of the rounds. Statements are displayed as used in the final survey round they were included. *No macroscopic metastases and negative cytology. PCI, Peritoneal Cancer Index.

### Protocol

Statements that reached consensus constitute a protocol to standardize the use and execution of staging laparoscopy in gastric cancer patients (see *supplementary material S6*). This includes indications for staging laparoscopy, when to assess local resectability, which structures to assess, how to score the burden of disease, how to perform peritoneal lavage, and when to biopsy. A template operation report was drafted to be used in clinical practice (see *supplementary material S7*).

## Discussion

This modified Delphi study established a consensus-based protocol for staging laparoscopy for gastric cancer patients. European surgeons reached consensus on key aspects such as indications for staging laparoscopy, details of intraoperative assessment, the preferred method for classifying the extent of peritoneal metastases, and peritoneal lavage technique. However, certain areas—such as the use of staging laparoscopy in patients undergoing primary surgical resection and the indication for re-laparoscopy—remained subject to debate. This protocol can be used as a stand-alone tool or to modify centres’ existing protocols. While it is acknowledged that prospective validation is necessary to assess compliance and clinical utility, implementation may facilitate data collection, improve comparability between centres, and promote international collaboration for trials concerning peritoneal metastases.

Lack of consensus on indications for staging laparoscopy is reflected by current guidelines. Most guidelines recommend staging laparoscopy in patients at high risk of peritoneal metastases, but lack specification of patient or disease-related criteria. For Siewert type II tumours, debate persists regarding whether an ‘intermediate’ risk of peritoneal metastases warrants staging laparoscopy. A national Delphi study was performed in the Netherlands, but failed to reach consensus on Siewert type II tumours^12^. In contrast, the present survey indicates that physicians can be guided by (PET-)CT and tumour characteristics to determine the indication for tumours of the junction. Tumours predominantly involving the stomach on (PET-)CT with high-risk features such as poorly cohesive cells or advanced tumour or nodal stage warrant staging laparoscopy. These features help in differentiating risk groups and are supported by recent studies.

The risk of peritoneal metastases seems to correlate with the extent of gastric infiltration of Siewert type II tumours, in particular when >4 cm of the stomach is involved^19^. Although limited evidence supports performing staging laparoscopy in early-stage tumours, diffuse-type and poorly cohesive tumours are subject to understaging in up to 40% of patients and confer a high risk of peritoneal dissemination, irrespective of cT and cN categories^20,21^. Moreover, endoscopic assessment of the extent of stomach infiltration of these tumours is challenging due to a diffuse growth pattern and submucosal infiltration^22^. Additionally, clinically differentiating T1–2 from T3–4 gastric tumours can be challenging. Endoscopic ultrasonography (EUS) may help to distinguish cT2 from cT3–4 disease and guide the decision to perform laparoscopy. However, EUS is not routinely recommended in European guidelines^23^. Although EUS shows a high sensitivity for cT1 tumours, its performance in differentiating cT2–4 lesions is inconsistent and does not clearly outperform diagnostic CT^24,25^. Hence, a pragmatic approach is to perform diagnostic laparoscopy when the tumour can be delineated on CT or if other high-risk features such as poorly cohesive tumours, including the endoscopic appearance of linitis plastica, or nodal involvement are present.

No consensus emerged on routinely assessing tumour resectability, despite reports indicating that approximately 1–4% of patients exhibit unresectable disease during staging laparoscopy^6,21,26^. Considering this percentage is relatively low, participants noted that complete assessment requires an extensive dissection, which carries risks and may complicate future surgery. Additionally, the resectability of a tumour may change during neoadjuvant therapy, though evidence for this is lacking. Therefore, selective assessment, guided by imaging, seems appropriate. Suspected infiltration preventing a radical resection requires further assessment. Importantly, if a tumour is located at the posterior wall of the stomach, opening the omental bursa is recommended. Although no consensus was reached, routine inspection of easily accessible structures such as the left liver lobe can be performed without additional risks.

Perioperative chemotherapy is the standard for curative treatment of gastric cancer in Europe^23^. However, approximately 10% of patients develop interval metastases after negative staging laparoscopy, often resulting in open-close surgery due to the low sensitivity of the restaging CT^6,26,27^. Restaging with laparoscopy has been proposed to address this issue, but, in the present study, there was no consensus on performing either a systematic or a global re-inspection. This was possibly the result of divided views, as both statements reached near 50% agreement. However, a minimally invasive approach allows for inspection with minimal trauma as long as frozen section is available. There was consensus though that restaging laparoscopy is necessary in case of suspected progression.

The role of routine peritoneal lavage is inadequately described in guidelines and remains unclear. The sensitivity of peritoneal fluid cytology is suboptimal and highly variable^28^. Although consensus was reached to perform lavage according to the AJCC staging manual, the present Delphi survey showed significant variability in surgeons’ opinions. While the location of lavage appears to affect detection rates, standardizing both the location and volume of instillation to improve sensitivity remains unproven. However, this does provide a baseline, facilitating studies on new detection methods for free cancer cells^29^. Literature on this issue is scarce, but techniques used for evaluation of free cancer cells differ between centres^8^. Conventional methods such as Papanicolaou, Giemsa, and haematoxylin and eosin staining yield a low sensitivity of approximately 60%. Novel techniques to detect free cancer cells include immunoassays, immunohistochemistry, reverse transcription PCR, and tumour-guided cell-free DNA analysis, and may offer improved sensivity^8,28,30^.

In European series, up to 12.5% of patients have free cancer cells without macroscopic lesions, but the clinical relevance of this diagnosis is subject to debate^6,21,31^. There was no consensus on peritoneal lavage in patients planned for gastrectomy without the need for staging laparoscopy. Participants cited logistical issues or a lack of consequences for treatment as reasons. Some treat patients with free cancer cells without peritoneal metastases with curative intent, despite the fact that guidelines classify it as M1 disease^23^. Several Asian studies report long-term survival, with 5-year survival up to 26% after chemotherapy and resection, but free cancer cells portend an equally poor prognosis when compared with macroscopic peritoneal disease in a European setting, underlining the prognostic relevance of free cancer cells^32–34^. A recent European consensus on metastatic gastric cancer management did not clarify optimal treatment or classification^35^. However, it concluded that conversion to negative cytology could be considered as oligometastatic disease (that is metastases limited to one organ) and may permit radical resection.

Strengths of this study include the predefined threshold of consensus, anonymity of results with iterative feedback, and recruitment of participants through organizational networks, allowing a group of up-to-date experts to be approached. However, the use of these networks prohibited calculation of the response rate and characterization of non-respondents for the first survey, potentially introducing bias. Restriction to European experts ensures homogeneity in patient populations and treatment approaches, but may limit its generalizability outside Europe. The incidence of gastric cancer is high in (East) Asia and there are differences in tumour biology, patient characteristics, and treatment paradigms between Eastern and Western countries. Screening programmes in Asia result in more early-stage disease detection. Therefore, most Asian countries do not perform staging laparoscopy as part of diagnostic workup, but rather as a tool to confirm eligibility in clinical trials, serving a different purpose^36,37^. It should be emphasized that the results of the present study are not evidence-based; they represent the collective view of a group of experts. Prospective validation is therefore necessary. Compliance with the proposed protocol should be assessed and it should be evaluated whether or not implementation of this consensus improves staging accuracy.

## Collaborators

Alexander A. F. A. Veenhof (Department of Surgery, Antoni van Leeuwenhoek, Amsterdam, The Netherlands); Adam Zeyara (Division of Oesophagogastric Surgery, Department of Surgery, Skåne University Hospital, Lund, Sweden); Alan Patrick Ainsworth (Department of Surgery, Odense University Hospital, Odense, Denmark); Alessia Malagnino (General and Emergency Surgery Department, Ospedale A. Manzoni Lecco, Lecco, Italy); Alexander W. Phillips (Northern Oesophagogastric Unit, Newcastle upon Tyne, UK); Anastasios Kottikias (General Oncological Hospital of Kifisia, Athens, Greece); Andrew R. Davies (Guys & St Thomas’ NHSFT, London, UK); Arto Kokkola (Department of Surgery, Helsinki University Hospital, Helsinki, Finland); Bo J. Noordman (Department of Surgery, Erasmus Medical Centre, Rotterdam, The Netherlands); Carlo Vallicelli (General, Emergency and Trauma Surgery, Maurizio Bufalini Hospital, Cesena, Italy); Carolina Canhoto (General Surgery Department, Centro Hospitalar Tondela-Viseu, Viseu, Portugal); Cecilia Monteiro (Instituto Português Oncologia Lisboa, Lisbon, Portugal); Claudia Neves Marques (Centro Hospitalar Universitário Lisboa Central, Lisbon, Portugal); Claudio Belluco (Department of Surgical Oncology, CRO Aviano National Cancer Institute IRCCS, Aviano, Italy); Daniel Reim (Department of Surgery, TUM School of Medicine and Health, Technical University of Munich, Munich, Germany); Daniele Marrelli (Unit of General Surgery and Surgical Oncology, Department of Medicine, Surgery and Neurosciences, University of Siena, Siena, Italy); Daromir Godula (First Department of Surgery, Jagiellonian University Medical College, Cracow, Poland); David James (Mitton Castle Hill Hospital, Hull University Teaching Hospitals, Hull, UK); Davide Zattoni (Ospedale Santa Maria delle Croci di Ravenna—AUSL Romagna, Ravenna, Italy); Dimitrios Schizas (First Department of Surgery, National and Kapodistrian University of Athens, Laikon General Hospital, Athens, Greece); Dimitrios Theodorou (Hippokration General Hospital, University of Athens, Athens, Greece); Dionysios Dellaportas (3rd Department of Surgery, NKUA, Attikon Hospital, Athens, Greece); Eelco B. Wassenaar (Department of Surgery, Gelre Hospitals, Apeldoorn, The Netherlands); Eider Talavera-Urquijo (Department of Surgery, University Hospital of Donostia, Donostia-San Sebastián, Spain); Fabio Uggeri (University of Milan-Bicocca IRCCSU Fondazione San Gerardo Monza, Monza, Italy); Fabrizio D’Acapito (General and Oncological Surgery, Morgagni-Pierantoni Hospital, AUSL Romagna, Forlì, Italy); Fausto Rosa (Università Cattolice del Sacro Cuore, Fondazione Policlinico Universitario A. Gemelli IRCCS, Rome, Italy); Federica Riccio (Department of Surgery, Oncology and Gastroenterology, University of Padua, Padua, Italy); Francesco Abboretti (Department of Visceral Surgery, Lausanne University Hospital (CHUV), Lausanne, Switzerland); Geert A. Simkens (Department of Surgery, Ziekenhuisgroep Twente, Almelo, The Netherlands) Gianluca Garulli (Chirurgia Generale e d’Urgenza Ospedale Infermi Rimini, Rimini, Italy); Gianmario Edoardo (Poto Università degli studi di Siena, Siena, Italy); Giovanni de Manzoni (Department of Surgery, University of Verona, Verona, Italy); Grard A. P. Nieuwenhuijzen (Department of Surgery, Catharina Hospital Eindhoven, Eindhoven, The Netherlands); Guido A. M. Tiberio (General Surgery, Department of Clinical and Experimental Sciences, University of Brescia at ASST Spedali Civili di Brescia, Brescia, Italy); Henk H. Hartgrink (Leiden University Medical Centre, Leiden, The Netherlands); Hanne Vanommeslaeghe (Department of Gastrointestinal Surgery, Ghent University Hospital, Ghent, Belgium); Hylke J. F. Brenkman (Department of Surgery, UMC Utrecht, Utrecht, The Netherlands); Ines Gockel (Department of Visceral, Transplant, Thoracic and Vascular Surgery, University Hospital of Leipzig, Leipzig, Germany); Ioannis G. Karavokyros (Medical School, National and Kapodistrian University of Athens, Athens, Greece); Ioannis Rouvelas (Department of Clinical Science, Intervention and Technology (CLINTEC), Division of Surgery and Oncology, Karolinska Institutet, and Department of Upper Abdominal Diseases, Karolinska University Hospital, Stockholm, Sweden); J. W. Haveman (University Medical Centre Groningen, University of Groningen, Groningen, The Netherlands); Jacopo Weindelmayer (Department of Surgery, University of Verona, Verona, Italy); Jan Willem T. Dekker (Department of Surgery, Reinier de Graaf Groep, Delft, The Netherlands); Jessie A. Elliott (Department of Surgery, Trinity St James’s Cancer Institute, Dublin, Ireland); Joos Heisterkamp (Department of Surgery, ETZ, Tilburg, The Netherlands); Jose A. L. Barbosa (Faculty of Medicine, University of Porto, Porto, Portugal); José P. Freire (ULSSM—HSM/FML, Hospital Santa Maria—Faculdade de Medicina de Lisboa, Lisbon, Portugal); Koen J. Hartemink (Department of Surgery, Antoni van Leeuwenhoek Hospital, Amsterdam, The Netherlands); Lapo Bencini (Department of Surgery, Careggi University Hospital, Florence, Italy); Lieven P. Depypere (Department of Thoracic Surgery, University Hospitals Leuven, Leuven, Belgium); Luca Provenzano (Università degli Studi di Padova U.O.C. Chirurgia Generale 1—Dipartimento di Scienze Chirurgiche Oncologiche e Gastroenterologiche, Padua, Italy); Lucia Moletta (Università degli Studi di Padova U.O.C. Chirurgia Generale 1—Dipartimento di Scienze Chirurgiche Oncologiche e Gastroenterologiche, Padua, Italy); Ludovico Carbone (Department of Medicine, Surgery and Neurosciences, University of Siena, Siena, Italy); Luigina Graziosi (University of Perugia, Perugia, Italy); Mark I. van Berge Henegouwen (Department of Surgery & Cancer Centre Amsterdam, Amsterdam UMC, University of Amsterdam, Amsterdam, The Netherlands); Magnus Nilsson (Division of Surgery and Oncology, CLINTEC, Karolinska Institutet, and Department of Upper Abdominal Diseases, Karolinska University Hospital, Karolinska, Sweden); Marcel A. Schneider (Department of Surgery, University Hospital of Zurich, Zurich, Switzerland); Marco Milone (University of Naples Federico II, Naples, Italy); Maria Bencivenga (General and Upper GI Surgery, University of Vetona, Vetona, Italy); Marloes Emous (Department of Surgery, MC Leeuwarden, Leeuwarden, The Netherlands); Mattia Berselli (General Surgery I, ASST Settelaghi, Varese, Italy); Mauro Zago (Department of General & Emergency Surgery—A. Manzoni Hospital—ASST Lecco, Lecco, Italy); Meindert N. Sosef (Department of Surgery, Zuyderland Medical Centre, Heerlen, The Netherlands); Michael Hareskov Larsen (Odense University Hospital, Odense, Denmark); Michele Manara (University of Milan, Milan, Italy); Michele Valmasoni (Department of Surgery, Oncology and Gastroenterology, Padova University Hospital, Padua, Italy); Michiel F. G. de Maat (Antwerp University Hospital, Antwerp, Belgium); Monica Gualtierotti (Division of Minimally Invasive Surgical Oncology, Niguarda Cancer Centre, ASST Grande Ospedale Metropolitano Niguarda, Milan, Italy); Monica Miro Martin (General Digestive Surgery Department, Bellvitge University Hospital, University of Barcelona-IDIBELL, L’Hospitalet de Llobregat, Spain); Fahad Mahmood (University Hospitals of North Midlands NHS Trust, Stoke, UK); Nezih Akkapulu (Department of General Surgery, Hacettepe University Hospital, Ankara, Turkey); Paolo Morgagni (Morgagni Pierantoni General Hospital Surgical Department, Forli, Italy); Paolo Parise (General Surgery Unit—Policlinico di Abano, Abano Terme, Italy); Paul A. Carroll (Galway University Hospital, Galway, Ireland); Paul M. Schneider (Digestive Oncology Tumour Centre, Hirslanden Medical Centre, Zurich, Switzerland); Pedro Azevedo Serralheiro (Centro Hospitalar e Universitário de Coimbra, Coimbra, Portugal) Peter Grimminger (University Medical Centre Mainz, Mainz, Germany); Ines Gockel (Department of Visceral, Transplant, Thoracic and Vascular Surgery, University Hospital of Leipzig, Leipzig, Germany); Radoslaw Pach (First Department of Surgery Jagiellonian University, Cracow, Poland); Raffaele De Luca (Department of Surgical Oncology, IRCCS Istituto Tumori ‘Giovanni Paolo II’, Bari, Italy); Renato Salvador (Department of Surgical, Oncological and Gastroenterological Sciences, University of Padova, Padua, Italy); Renol M. Koshy (Leicester Royal Infirmary, Leicester, UK); Rita Alfieri (Upper Gastrointestinal Surgery Unit, IRCCS Humanitas Research Hospital, Rozzano, Milan, Italy); Sara Pollesel (IRCCS CRO Aviano, Pordenone, Italy); Markus Schäfer (Department of Visceral Surgery, University Hospital of Lausanne, Lausanne, Switzerland); Sheraz R. Markar (Nuffield Department of Surgery, University of Oxford, Oxford, UK); Silvia Ministrini (Università degli studi di Brescia, Brescia, Italy); Stefan Antonowicz (Imperial College London, London, UK); Stefania A. Piccioni (Unit of General Surgery and Surgical Oncology, Department of Medicine, Surgery and Neurosciences, University of Siena, Siena, Italy); Stefano Olmi (Università Viata e Salute, Policlinico San Marco, Zingonia (BG), Italy); Stefano Rausei (Department of Surgery, ASST Settelaghi, Varese, Italy); Styliani Mantziari (Lausanne University Hospital, Lausanne, Switzerland); Tania Triantafyllou (Department of Surgery, Hippocration General Hospital of Athens, University of Athens, Athens, Greece); Victor Turrado-Rodriguez (Unit of Oesophagogastric Surgery, General and Digestive Surgery Department, Clínic Barcelona, Barcelona, Spain); Wendy Kelder (Department of Surgery, Martini Hospital, Groningen, The Netherlands); Yannick Deswysen (Cliniques Universitaires Saint-Luc, Brussels, Belgium); Yves Borbély (Department of Visceral Surgery and Medicine, Inselspital, Bern University Hospital, Bern, Switzerland).

## Supplementary Material

znaf144_Supplementary_Data

## Data Availability

The data that support the findings of this study are available from the corresponding author upon reasonable request.
