# Supplementary material for: Staging laparoscopy for gastric cancer: European consensus
Source: Br J Surg. 2025 Sep 5;112(9):znaf144. doi: 10.1093/bjs/znaf144 (PMC12412212; doi:10.1093/bjs/znaf144)
Supplement: znaf144_Supplementary_Data [file znaf144_supplementary_data.docx]

**Supplementary material**

**Staging laparoscopy for gastric cancer: a European consensus**

*Members:* Sander JM van Hootegem^1^, Niels AD Guchelaar^2^, Karen van der Sluis^3^, Lianne Triemstra^4^, Stefan P Mönig^5^, Karol Rawicz-Pruszyński^6^, Riccardo Rosati^7^, Paolo Morgagni^8^, Maria Erodotou^1^, Leonardo Solaini^8^ , Giovanni De Manzoni^9^, Wojciech Polkowski^6^, Francesco Pucetti^7^, Simone Giacopuzzi^9^, Suzanne S Gisbertz^10^, Jimmy BY So^11^, Jelle Ruurda^4^, Pieter SL van der Sluis^1^, Sjoerd M Lagarde^1^, Johanna W van Sandick^3^, Bas PL Wijnhoven^1^, Collaborators †

^1^ Department of Surgery, Erasmus Medical Center, Rotterdam, The Netherlands

^2^ Department of Medical Oncology, Erasmus MC Cancer Institute, Rotterdam, the Netherlands

^3^ Department of Surgery, Antoni van Leeuwenhoek, Amsterdam, the Netherlands

^4^ Department of Surgery, University Medical Center Utrecht, Utrecht, the Netherlands

^5^ Upper-GI-Surgery, University Hospital of Geneva, Geneva, Switzerland

^6^ Department of Surgical Oncology, Medical University of Lublin, Lublin, Poland

^7^ Department of Gastrointestinal Surgery, IRCCS San Raffaele Hospital, Milan, Italy

^8^ Department of General Surgery, Morgagni-Pierantoni Hospital, Forlì, Italy

^9^ Upper G.I. Surgery Division, University of Verona, Verona, Italy

^10^ Department of Surgery, Amsterdam UMC location University of Amsterdam, Amsterdam, The Netherlands

^11^ Department of Surgery, Yong Loo Lin School of Medicine, National University of Singapore, Singapore, Singapore

† Listed under the header ‘Collaborators’ in Supplement 1.

*Correspondence to:* Professor BPL Wijnhoven mail: b.wijnhoven@erasmusmc.nl

**Index**

***S1.*** List of collaborators page 2-4

***S2.*** Removed statements page 5

***S3.*** Overview of guidelines page 6

***S4.*** Supplementary tables and figures page 7

***S5.*** Inventory assessment of cytological processing in steering committee centers page 8

***S6.*** Template for standardized operation report page 9

***S7.*** Summary of results page 10

**S1.** *List of collaborators*

| Author name | Affiliation |
| --- | --- |
| Alexander A.F.A. Veenhof | Department of Surgery, Antoni van Leeuwenhoek, Amsterdam, the Netherlands |
| Adam Zeyara | Division of Esophagogastric Surgery, Department of Surgery, Skåne University Hospital, Lund, Sweden |
| Alan Patrick Ainsworth | Department of Surgery, Odense University Hospital, Odense, Denmark |
| Alessia Malagnino | General and Emergency Surgery Department, Ospedale A. Manzoni Lecco, Lecco, Italy |
| Alexander W. Phillips | Northern Oesophagogastric Unit, Newcastle upon Tyne, United Kingdom |
| Anastasios Kottikias | General Oncological Hospital of Kifisia, Athens, Greece |
| Andrew R. Davies | Guys & St Thomas’ NHSFT, London, United Kingdom |
| Arto Kokkola | Department of Surgery, Helsinki University Hospital, Helsinki, Finland |
| Bo.J. Noordman | Department of Surgery, Erasmus Medical Center Rotterdam, The Netherlands |
| Carlo Vallicelli | General, Emergency and Trauma Surgery, Maurizio Bufalini Hospital, Cesena, Italy |
| Carolina Canhoto | General Surgery Department, Centro Hospitalar Tondela-Viseu, Viseu, Portugal |
| Cecilia Monteiro | Instituto Português Oncologia Lisboa, Lisbon, Portugal |
| Claudia Neves Marques | Centro Hospitalar Universitário Lisboa Central, Lisbon, Portugal |
| Claudio Belluco | Department of Surgical Oncology, CRO Aviano National Cancer Institute IRCCS, Aviano, Italy |
| Daniel Reim | Department of Surgery, Technical University of Munich, TUM School of Medicine and Health, Munich, Germany |
| Daniele Marrelli | Unit of General Surgery and Surgical Oncology, Department of Medicine, Surgery and Neurosciences, University of Siena, Siena, Italy |
| Daromir Godula | First Department of Surgery, Jagiellonian University Medical College, Cracow, Poland |
| David James Mitton | Castle Hill Hospital, Hull University Teaching Hospitals, Hull, United Kingdom |
| Davide Zattoni | Ospedale Santa Maria delle Croci di Ravenna - AUSL Romagna, Ravenna, Italy |
| Dimitrios Schizas | First Department of Surgery, National and Kapodistrian University of Athens, Laikon General Hospital, Athens, Greece |
| Dimitrios Theodorou | Hippokration General Hospital, University of Athens, Athens, Greece |
| Dionysios Dellaportas | 3rd Department of Surgery, NKUA, Attikon Hospital, Athens, Greece |
| Eelco B. Wassenaar | Department of Surgery, Gelre Hospitals, Apeldoorn, the Netherlands |
| Eider Talavera-Urquijo | Department of Surgery, University Hospital of Donostia, Donostia-San Sebastián, Spain |
| Fabio Uggeri | University of Milan-Bicocca IRCCSU fondazione San Gerardo Monza, Monza, Italy |
| Fabrizio D'Acapito | General and Oncologic Surgery, Morgagni-Pierantoni Hospital, AUSL Romagna, Forlì, Italy |
| Fausto Rosa | Università Cattolice del Sacro Cuore, Fondazione Policlinico Universitario A. Gemelli IRCCS, Rome, Italy |
| Federica Riccio | Department of Surgery, Oncology and Gastroenterology, University of Padua, Padua, Italy |
| Francesco Abboretti | Department of Visceral Surgery, Lausanne University Hospital (CHUV), Lausanne, Switzerland |
| Geert A. Simkens | Department of Surgery, Ziekenhuisgroep Twente, Almelo, the Netherlands |
| Gianluca Garulli | Chirurgia Generale e d’Urgenza Ospedale Infermi Rimini, Rimini, Italy |
| Gianmario Edoardo Poto | Università degli studi di Siena, Siena, italy |
| Giovanni de Manzoni | Deptartment of Surgery, University of Verona, Verona, Italy |
| Grard A.P. Nieuwenhuijzen | Department of Surgery, Catharina Hospital Eindhoven, Eindhoven, The Netherlands |
| Guido A.M. Tiberio | General Surgery, Department of Clinical and Experimental Sciences, University of Brescia at ASST Spedali Civili di Brescia, Brescia, Italy |
| Henk H. Hartgrink | Leiden University Medical Center, Leiden, The Netherlands |
| Hanne Vanommeslaeghe | Department of Gastrointestinal Surgery, Ghent University Hospital, Ghent, Belgium |
| Hylke J.F. Brenkman | Department of Surgery, UMC Utrecht, Utrecht, the Netherlands |
| Ines Gockel | Department of Visceral, Transplant, Thoracic and Vascular Surgery, University Hospital of Leipzig, Leipzig, Germany |
| Ioannis G. Karavokyros | Medical School, National ans Kapodistrian University of Athens, Athens, Greece |
| Ioannis Rouvelas | Department of Clinical Science, Intervention and Technology (CLINTEC), Division of Surgery and Oncology, Karolinska Institutet, and Department of Upper Abdominal Diseases, Karolinska University Hospital, Stockholm, Sweden |
| J.W. Haveman | University Medical Center Groningen, University of Groningen, Groningen, the Netherlands |
| Jacopo Weindelmayer | Deptartment of Surgery, University of Verona, Verona, Italy |
| Jan Willem T. Dekker | Department of Surgery, Reinier de Graaf Groep, Delft, the Netherlands |
| Jessie A. Elliott | Department of Surgery, Trinity St. James's Cancer Institute, Dublin, Ireland |
| Joos Heisterkamp | Departement of Surgery, ETZ, Tilburg, The Netherlands |
| Jose A.L. Barbosa | Faculty of Medicine, University of Porto, Porto, Portugal |
| José P. Freire | ULSSM - HSM/FML, Hospital Santa Maria - Faculdade de Medicina de Lisboa, Lisbon, Portugal |
| Koen J. Hartemink | Department of Surgery, Antoni van Leeuwenhoek Hospital, Amsterdam, The Netherlands |
| Lapo Bencini | Department of Surgery, Careggi University Hospital, Florence, Italy |
| Lieven P. Depypere | Department of Thoracic Surgery, University hospitals Leuven, Leuven, Belgium |
| Luca Provenzano | Università degli Studi di Padova U.O.C. Chirurgia Generale 1 - Dipartimento di Scienze Chirurgiche Oncologiche e Gastroenterologiche, Padua, Italy |
| Lucia Moletta | Università degli Studi di Padova U.O.C. Chirurgia Generale 1 - Dipartimento di Scienze Chirurgiche Oncologiche e Gastroenterologiche, Padua, Italy |
| Ludovico Carbone | Department of Medicine, Surgery and Neurosciences, University of Siena, Siena, Italy |
| Luigina Graziosi | University of Perugia, Perugia, Italy |
| Mark I. van Berge Henegouwen | Department of Surgery & Cancer Center Amsterdam, Amsterdam UMC, University of Amsterdam, Amsterdam, the Netherlands |
| Magnus Nilsson | Division of Surgery and Oncology, CLINTEC, Karolinska Institutet and Department of Upper Abdominal Diseases, Karolinska University Hospital, Karolinska, Sweden |
| Marcel A. Schneider | Department of Surgery, University Hospital of Zurich, Zurich, Switzerland |
| Marco Milone | University of Naples Federico II, Naples, Italy |
| Maria Bencivenga | General and Upper GI Surgery, University of Vetona, Vetona, Italy |
| Marloes Emous | Department of Surgery, MC Leeuwarden, Leeuwarden, the Netherlands |
| Mattia Berselli | General Surgery I, ASST Settelaghi, Varese, Italy |
| Mauro Zago | Dept. General & Emergency Surgery - A. Manzoni Hospital - ASST Lecco, Lecco, Italy |
| Meindert N. Sosef | Department of Surgery, Zuyderland Medical Center, Heerlen, the Netherlands |
| Michael Hareskov Larsen | Odense University Hospital, Odense, Denmark |
| Michele Manara | University of Milan, Milan, Italy |
| Michele Valmasoni | Padova University Hospital, Department of Surgery, Oncology and Gastroenterology, Padua, Italy |
| Michiel F.G. de Maat | Antwerp University Hospital, Antwerp, Belgium |
| Monica Gualtierotti | Division of Minimally Invasive Surgical Oncology, Niguarda Cancer Center, ASST Grande Ospedale Metropolitano Niguarda, Milan, Italy |
| Monica Miro Martin | General Digestive Surgery Department, Bellvitge University Hospital, University of Barcelona-IDIBELL, L'Hospitalet de Llobregat, Spain |
| Fahad Mahmood | University Hospitals of North Midlands NHS Trust, Stoke, United Kingdom |
| Nezih Akkapulu | Department of General Surgery, Hacettepe University Hospital, Ankara, Turkey |
| Paolo Morgagni | Morgagni Pierantoni General Hospital Surgical Department, Forli, Italy |
| Paolo Parise | General Surgery Unit - Policlinico di Abano, Abano Terme, Italy |
| Paul A. Carroll | Galway University Hospital, Galway, Ireland |
| Paul M. Schneider | Digestive Oncology Tumor Center, Hirslanden Medical Center, Zurich, Switzerland |
| Pedro Azevedo Serralheiro | Centro Hospitalar e Universitário de Coimbra, Coimbra, Portugal |
| Peter Grimminger | University Medical Center Mainz, Mainz, Germany |
| Ines Gockel | Department of Visceral, Transplant, Thoracic and Vascular Surgery, University Hospital of Leipzig, Leipzig, Germany |
| Radoslaw Pach | First Department of Surgery Jagiellonian University, Cracow, Poland |
| Raffaele De Luca | Department of Surgical Oncology IRCCS Istituto Tumori " Giovanni Paolo II", Bari, Italy |
| Renato Salvador | University of Padova, Italy. Department of Surgical, Oncological and Gastroenterological Sciences, Padua, Italy |
| Renol M. Koshy | Leicester Royal Infirmary, Leicester, United Kingdom |
| Rita Alfieri | Upper Gastrointestinal Surgery Unit, IRCCS Humanitas Research Hospital, Rozzano, Milan, Italy |
| Sara Pollesel | IRCCS CRO Aviano, Pordenone, Italy |
| Markus Schäfer | Dep. of Visceral Surgery, University Hospital of Lausanne, Lausanne, Switzerland |
| Sheraz R. Markar | Nuffield Department of Surgery, University of Oxford, Oxford, United Kingdom |
| Silvia Ministrini | Università degli studi di Brescia, Brescia, Italy |
| Stefan Antonowicz | Imperial College London, London, United Kingdom |
| Stefania A. Piccioni | Unit of General Surgery and Surgical Oncology, Department of Medicine, Surgery and Neurosciences, University of Siena, Siena, Italy |
| Stefano Olmi | Università Viata e Salute, Policlinico San Marco, Zingonia (BG), Italy |
| Stefano Rausei | Department of Surgery, ASST Settelaghi, Varese, Italy |
| Styliani Mantziari | Lausanne University Hospital, Lausanne, Switzerland |
| Tania Triantafyllou | Department of Surgery,Hippocration General Hospital of Athens,University of Athens, Athens, Greece |
| Victor Turrado-Rodriguez | Unit of Esophagogastric Surgery, General and Digestive Surgery Department, Clínic Barcelona, Barcelona, Spain |
| Wendy Kelder | Department of Surgery, Martini Hospital, Groningen, The Netherlands |
| Yannick Deswysen | Cliniques Universitaires Saint-Luc, Brussels, Belgium |
| Yves Borbély | Department of Visceral Surgery and Medicine, Inselspital, Bern University Hospital, Bern, Switzerland |

**S2.** Statements that were removed after the first survey.

| ***Statement*** |  |  |  | **Reasons** | |
| --- | --- | --- | --- | --- | --- |
| 1. *Resectability regarding infiltration of the esophagus and/or diaphragm should be assessed.* | | | | | - Different views on whether resectability should be assessed routinely or only when suspect for infiltration on imaging - Risks and difficulty depends on the extent of dissection required - Some believe it is more appropriate to assess after neoadjuvant therapy as this may change tumor resectability |
| 1. *Resectability regarding infiltration of the liver should be assessed.* | | | | |  |
| 1. *Resectability regarding infiltration of the pancreas, vascular structures posterior of the stomach and the retroperitoneum should be assessed.* | | | | |  |
| 1. *Resectability regarding infiltration of the liver should be assessed by standard.* | | | | |  |
| 1. *Resectability regarding infiltration of the mesocolon should be assessed.* | | | | |  |
| 1. *Resectability regarding infiltration of the spleen should be assessed.* | | | | |  |

**S3.** Overview of guidelines

| **Organisation** | **Region (year)** | **Tumor location** | **Characteristics/stage** | **Scoring system** | **Recommendations on cytology** |
| --- | --- | --- | --- | --- | --- |
| ESMO | Europe (2022) | Resectable gastric cancer | All stage IB-III (greater benefit in cT3-4 and poorly cohesive) | PCI | To perform routinely |
| FMS | The Netherlands  (2023) | Potentially curable gastric cancer | cT3-4 and/or cN+ | - | - |
| S3 | Germany  (2019) | Gastric cancer  or junctional cancer (Siewert II and III) | cT3-4 (when neoadjuvant chemotherapy is indicated) | PCI | To perform routinely |
| GIRCG | Italy  (2015) | Gastric cancer | Patients at risk of peritoneal carc. or suspect on CT | - | Useful completion of staging |
| AUGIS | UK (2024) | Gastric cancer or  esophageal cancer extending below the diaphragm | - | - | - |
| NICE | UK (2023) | Potentially curable gastric cancer or  esophageal and junctional cancer if it will help guide management | - | - | - |
| SEOM | Spain (2019) | Resectable gastric cancer | cT3-4, poor tumor diff., and/or high nodal burden | - | May help identify occult carcinomatosis |
| French Intergroup | France (2017) | Optional in resectable gastric cancer | - | - | To perform routinely |
| Swedish National Guidelines | Sweden (2022) | Gastric cancer | cT3-4, diffuse type, and suspicion of peritoneal disease (or ascites) on CT | - | Can be performed |
| **Non-European guidelines** | | | | | |
| JGCA | Japan (2021) | Gastric cancer | Advanced (weakly recommended) | - | - |
| SAGES | USA (2010) | Gastric cancer | cT3-4 without evidence of lymph node or distant metastasis on imaging | - | - |
| NCCN | USA (2022) | Resectable gastric cancer | ≥cT1b or higher when considering preoperative chemoradiation and/or surgery | - | To perform routinely |

**S4.** Supplementary figures/tables

**Supplementary figure 1A-B** Questions on preferred site (A) and amount of instillation (B) for peritoneal lavage

**
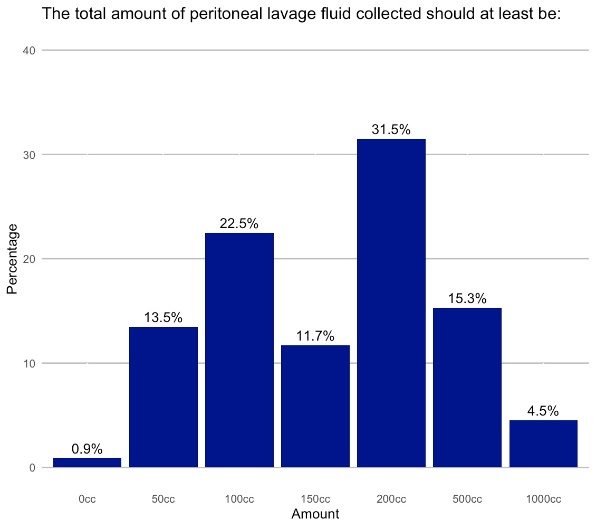
 A B**

| **Region** | **Percentage (multiple answers possible)** |
| --- | --- |
| Left upper abdomen | 72.1% |
| Right upper abdomen | 66.7% |
| Pouch of Douglas | 58.6% |
| Over the gastric tumor | 37.8% |
| Entire abdomen | 27.8% |

**S5.** Inventory assessment of cytological processing in steering committee centers

| **Question** | **Answers (n = 7)** |
| --- | --- |
| Peritoneal lavage fluid that is taken from different locations in the abdomen should be sent in and analysed separately for presence of free cancer cells. | - Yes (3) - No (2) - Differs (2) |
| When both ascites and peritoneal washings are collected, they are/should analysed for free cancer cells separately. | - Yes (7) |
| The minimum amount of peritoneal washing or ascites for cytological assessment in our centre is: | - No minimum (1) - 20cc (1) - 50cc (3) - 500cc (2) |
| The standard technique(s) (first assessment) used for cytological assessment of peritoneal washings in our centre is/are: | - Papanicolaou staining and May-Grunwald-Giemsa staining (2) - Hemacolor and Papnicolaou staining (1) - Papanicolaou + May-Grunwald-Giemsa staining or H&E (1) - Hematoxylin and eosin staining (1) - Immunohistochemistry (1) |
| What technique(s) is/are used when there is doubt about the presence of cancer cells after the standard assessment? | - Cell block with immunohistochemistry (4) - Muycarmine staining (1) - No other techniques used (1) |

**S6.** *Template for standardized operation report for staging laparoscopy in gastric cancer*

| **Operation:** | Staging laparoscopy |
| --- | --- |
| **Indication:** | Gastric tumor located in the **(fundus/corpus/antrum/pylorus)** at the **(anterior/posterior wall or greater/lesser curvature)**  Junctional **(Siewert II/Siewert III)** tumor  Stage: **cT…N…M…**  Poorly cohesive: **yes/no**  Radiological suspicion of peritoneal metastases:  **yes/no**  Radiological suspicion of infiltration: **no/yes (in the ….)** |
| **Surgeon:** |  |
| **Report** | |
| General | Time-out procedure, general anesthesia, disinfection, and sterile covering. (..) |
|  | Open introduction **no abnormalities/abnormalities.**  Inspection of the stomach **no abnormalities/abnormalities.**  The tumor is localized in the **…**  Inspection of the tumor **no abnormalities/abnormalities.**  Serosal tumor involvement **yes/no.** |
| Resectability | *Fill in on indication*: based on preoperative imaging; the … was/were assessed on ingrowth and showed **abnormalities/no abnormalities**.  The lesser sec was **opened/not opened**. |
| PCI score | **Presence/absence** of peritoneal metastases according to the Peritoneum Cancer Index (PCI):  0 Central =  1 Right Upper =  2 Epigastrium =  3 Left Upper =  4 Left Flank =  5 Left Lower =  6 Pelvis =  7 Right Lower =  8 Right Flank =  9 Upper jejunum =  10 Lower Jejunum =  11 Upper Ileum =  12 Lower Ileum =  Total PCI score = |
| Peritoneal cavity | Inspection of the greater omentum **no abnormalities/abnormalities.**  Inspection of the lesser omentum **no abnormalities/abnormalities.**  Inspection of the mesentery of the small intestine **no abnormalities/abnormalities.**  Inspection of the hepatoduodenal ligament **no abnormalities/abnormalities.**  Inspection of the Douglas cavity **no abnormalities/abnormalities.**  Inspection of the ovaries **no abnormalities/abnormalities.** |
| Peritoneal lavage | Ascites **present/absent. … cc** ascites was aspirated.  Peritoneal washings **…cc** in left and right subphrenic space and Douglas cavity. |
| Biopsy | … biopsies were taken from region(s) … |
| Other findings | Adhesions **present/absent** in …  Other relevant findings: … |
| **Conclusion** | Staging laparoscopy, **resectable/non-resectable/resectability was not assessed**.  PCI: …, cytology performed, … biopsies taken. |

**S7.** *Summary of consensus*

| **Domain** | **Details** | |
| --- | --- | --- |
| Indications | **Tumor characteristics** | Perform a staging laparoscopy in patients with (gastric) carcinoma with high risk of peritoneal metastases, which include:   - cT3-4 - cN+ - Poorly cohesive tumors - Radiological suspicion of peritoneal dissemination |
|  | **Tumor location** | - Siewert Type I: Staging laparoscopy not required. - Siewert Type II: Staging laparoscopy indicated if endoscopy and PET-CT show predominant stomach involvement; decision also based on tumor characteristics. - Siewert Type III: Staging laparoscopy required. |
| Resectability | - No consensus to perform assess routinely - Specific structures requiring surgical dissection (e.g., esophagus, pancreas) should be assessed only if suspected for involvement based on imaging. - The omental bursa (lesser sac) should be opened to assess infiltration if the tumor is located posteriorly on the stomach. | |
| Inspection of peritoneal cavity | - Perform systematic inspection and documentation using the Peritoneum Cancer Index (PCI). - Structures that should at least be assessed: the greater and lesser omentum, (mesentery of the) small intestine, hepatoduodenal ligament, pelvis, Douglas pouch, and ovaries | |
| Peritoneal lavage and biopsy | **Peritoneal lavage** | - Perform peritoneal lavage for cytological assessment in all patients who undergo a staging laparoscopy. - Collect free fluid (ascites) if present; if less than 50cc is aspirated, perform lavage. - Perform peritoneal lavage according to the AJCC staging manual:   - Lavage sites: upper right and left subphrenic spaces and Douglas pouch.   - Instillation and aspiration: instill at least 200cc, aspirating a minimum of 50cc for cytology. |
|  | **Biopsy** | - Biopsy not required for all suspected peritoneal abnormalities, but disease should be confirmed in at least one region.   - For limited disease: biopsy all regions.   - For extensive disease: one region is sufficient unless taking multiple biopsies will impact treatment decisions. |
| Re-laparoscopy | - Indicated in case of tumor progression, but no consensus to perform routinely. - Not necessarily planned separately; depends on logistical planning. | |
